# INTEGRATED PERSON-CENTERED INTERVENTIONS FOR OLDER PEOPLE’S CARE IN SWEDEN: SCOPING REVIEW AND EXPERT OPINION

**DOI:** 10.1093/geroni/igae098.2636

**Published:** 2024-12-31

**Authors:** Mariam Kirvalidze, Anne-Marie Boström, Anne Liljas, Megan Doheny, Anne Hendry, Brendan McCormack, Janne Agerholm, Amaia Calderón-Larrañaga

**Affiliations:** Karolinska Institutet, Stockholm, Stockholms Lan, Sweden; Karolinska Institutet, Stockholm, Stockholms Lan, Sweden; Karolinska Institutet, Stockholm, Stockholms Lan, Sweden; Karolinska Institutet, Stockholm, Stockholms Lan, Sweden; University of the West of Scotland, Paisley, Scotland, United Kingdom; University of Sydney, Sydney, New South Wales, Australia; Karolinska Institutet, Stockholm, Stockholms Lan, Sweden; Karolinska Institutet, Stockholm, Stockholms Lan, Sweden

## Abstract

The recent shift towards a holistic, person-centered approach to older people’s care that considers a person’s overall well-being requires collaboration within and between medical and social healthcare organizations. To map and contextualize the scientific literature on the effectiveness of integrated person-centered care interventions, including implementation experiences in Sweden. Publications were identified by searching MEDLINE and contacting experts. Workshop discussions in Stockholm (Sweden), on 21/03/2023, were recorded and transcribed. Original peer-reviewed articles published in Swedish or English from 01/01/2000 to 10/03/2023, were screened. Evidence from included studies was narratively synthesized and interventions were categorized using two frameworks: (1) Integrated Care for Older People (ICOPE) and (2) Integrated People Centered Health Services (IPCHS). Workshop discussions were thematically summarized. We included 73 publications analyzing 31 unique and heterogeneous interventions pertaining, mainly, to the micro and meso levels. Among publications measuring mortality, 15% were effective. Subjective health outcomes showed improvement in 24% of publications, morbidity outcomes in 42%, disability in 48%, and service utilization in 58%. Experts emphasized the need for participatory designs and shared ownership for adoption and scalability of interventions, and transitions from person- to people-centered integrated care. In conclusion, integrated person-centered care interventions demonstrated the strongest evidence for the optimization of service utilization. Unexpectedly, evidence for health outcomes was less consistent. This could be due to several issues mentioned by the experts, such as poor evaluation practices, lack of participatory research approaches, and scarcity of macro-level initiatives. Our findings offer internationally transferable perspectives on the real-world challenges in delivering integrated care.

